# Study on the adsorption performance of modified high silica fly ash for methylene blue

**DOI:** 10.1039/d4ra04017a

**Published:** 2024-07-08

**Authors:** Xuying Guo, Zilong Zhao, Xinle Gao, Yanrong Dong, Honglei Fu, Xiaoyue Zhang

**Affiliations:** a College of Science, Liaoning Technical University Fuxin 123000 Liaoning China guoxuying@lntu.edu.cn; b College of Mining, Liaoning Technical University Fuxin 123000 Liaoning China; c College of Civil Engineering, Liaoning Technical University Fuxin 123000 Liaoning China

## Abstract

Presently, there are several issues associated with solid waste fly ash, such as its accumulation and storage, low comprehensive utilization rate, lack of high-value utilization technology, environmental risk and ecological impact. Thus, based on the high silica content and adsorption characteristics of fly ash, two novel adsorbents, namely mesoporous silica-based material (MSM) and sodium dodecyl sulfate-modified fly ash (SDS-FA), were prepared using an ultrasound-assisted alkali fusion-hydrothermal method and surface modification method. Furthermore, effects of adsorbent dosage, initial pH, contact time, and initial concentration of the solution on the adsorption of the organic pollutant methylene blue (MB) by fly ash, MSM, and SDS-FA were investigated to select the optimal modified high silica fly ash adsorbent. Based on the adsorption isotherms and adsorption kinetics, together with SEM, XRD, FTIR and BET analyses, the adsorption mechanism of MSM for MB was revealed. The results showed that under the conditions of an adsorbent dosage of 2 g L^−1^, initial pH of 9, contact time of 150 min, and initial concentration of 100 mg L^−1^, MSM and SDS-FA exhibited removal efficiencies of 92.69% and 84.64% for MB, respectively, which were significantly higher than that of fly ash alone. The adsorption of MB by MSM and SDS-FA followed the Langmuir model and pseudo-second-order kinetics, indicating monolayer adsorption with chemical adsorption as the dominant mechanism. The mechanism of the adsorption of MB by MSM is mainly the result of the synergistic effect among its increased specific surface area, hydrogen bonding, ion exchange, and electrostatic interactions. After five cycles of adsorption–desorption process, the removal efficiency of MSM for MB consistently remained above 80%. Therefore, MSM can serve as a valuable reference for the resource utilization of fly ash and remediation of dye-polluted wastewater.

## Abstract 2

Based on the high silica content and adsorption characteristics of fly ash, two novel adsorbents, namely mesoporous silica-based material (MSM) and sodium dodecyl sulfate-modified fly ash (SDS-FA), were prepared.

## Graphical abstract

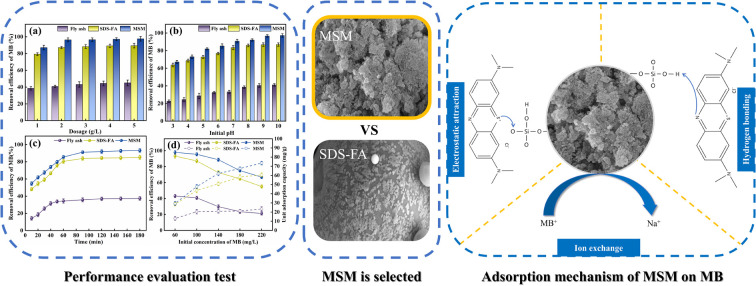

## 1 Introduction

With the rapid development of the industrial economy, the demand for coal and its total consumption have been increasing. Consequently, the production and storage of the coal combustion by-product fly ash have been increasing annually. Fly ash is primarily used in the production of cement, road construction, and mineral extraction; however, its comprehensive utilization has not witnessed significant changes in recent years.^1–3^ Against the backdrop of the “dual carbon” strategy, the coal industry in China urgently requires the transformative development of the comprehensive utilization of solid waste resources.^4–7^ Therefore, exploring economic, green, and sustainable utilization technologies for fly ash has become an urgent issue.

In recent years, many researchers have focused on the utilization of fly ash in environmental pollution remediation due to its porous structure, high adsorption activity, and other characteristics. Research has been dedicated to employing fly ash as a “waste-to-treat-waste” approach for the removal of heavy metals, organic compounds, dyes, and other pollutants in the water environment.^8,9^ However, the effectiveness of using untreated fly ash for pollutant removal is limited by its lack of surface functional groups and poor selectivity. Therefore, physical and chemical modifications are often necessary to enhance the activity of fly ash.^10–12^ In this case, high-temperature modification technology has advantages such as high volume reduction rate and low leaching rate of heavy metals. However, it has high energy consumption and the subsequent treatment of volatile heavy metals such as Pb, Cd, and Zn in the flue gas incurs higher costs.^13^ Hussain *et al.*^14^ utilized NaOH and HCl to modify fly ash and employed it for the treatment of the dyes Direct Fast Scarlet 4BS and Direct Sky Blue 5B. Under the optimal conditions, the maximum removal efficiency reached 96.03% and 93.82%, respectively. However, the acid–alkali modification required strict control of the conditions such as acid–alkali concentration, given that improper conditions can reduce the activity of silica, making it difficult to enhance the activity of fly ash. Chen *et al.*^15^ synthesized the NaP1 zeolite *via* the hydrothermal method for the degradation of methylene blue. Under the conditions of 180 °C, 1 h, 0.5 g Na_2_SiO_3_, and 10 mL water, the removal efficiency reached 96% within 12 h, exceeding the removal efficiency of fly ash (38%). However, the synthesis of zeolites from fly ash typically requires a long reaction time and results in relatively low purity. Açışlı *et al.*^16^ conducted the surface modification of fly ash using cetyltrimethylammonium bromide (CTAB), and the results showed that the modified fly ash effectively removed the anionic dye Acid Blue 185. Surface modification methods are simple, flexible, and require small amounts of modifiers. Furthermore, by introducing specific functional groups for targeted modification, the selective adsorption of pollutants can be achieved. However, the selection of surface modifiers can be challenging and improper handling can lead to the agglomeration of fly ash. In summary, in terms of activating the activity of fly ash, the modification methods can be ranked as follows: high-temperature modification < acid–alkali modification < hydrothermal modification < surface modification. Additionally, these traditional modification methods have disadvantages such as high treatment costs, complex processes, and instability. Therefore, it is worth considering the use of fly ash as a substrate to prepare new environmentally friendly and efficient adsorbent materials.

Currently, mesoporous silica-based materials are widely used as adsorbents and catalyst supports in environmental remediation due to their regular and ordered pore structure, narrow pore size distribution, and tunable pore size.^17^ The conventional methods for the synthesis of mesoporous silica-based materials mainly include hydrothermal methods using silica aerosol or tetraethyl orthosilicate (TEOS) as the silicon source. However, these methods suffer from high raw material costs and low resource utilization efficiency.^18^ Therefore, the exploration of methods for the synthesis for mesoporous silica-based materials should focus on finding more cost-effective silicon source materials and developing simpler preparation processes. Yang *et al.*^19^ synthesized mesoporous silica (MS) by reacting coal ash with CO_2_, and the results showed that MS had an adsorption capacity of 106.2 mg g^−1^ for phenol. Dong *et al.*^20^ prepared mesoporous zinc silicate composite materials using hydrothermal methods with waste iron tailings as the raw material. The results demonstrated that the efficiency for the removal of methylene blue exceeded 97% within 10 min and the maximum adsorption capacity was 180.5 mg g^−1^, which is more than nine times that of the original iron tailings. This demonstrates the potential of industrial solid waste as a substitute for the toxic organic silicon. Bukhari *et al.*^21^ studied the effect of ultrasonic energy on the zeolitization of the aluminum silicate components in fly ash at different temperatures. They found that ultrasound assistance could accelerate the heterogeneous reaction between the liquid and solid reactants, leading to a decrease in crystallization time and temperature. Therefore, the use of high-silicon fly ash as a silicon source, combined with ultrasound-assisted and alkali fusion-hydrothermal methods is considered feasible for the development of green methods for the synthesis of mesoporous silica-based materials. Compared to mesoporous silica-based materials, materials modified with sodium dodecyl sulfate (SDS) as a surface modifier have also been widely studied. Zhang *et al.*^22^ used SDS to modify activated carbon for the removal of PAH4 from edible oil. The modified activated carbon showed a promising adsorption performance for polycyclic aromatic hydrocarbons in edible oil. Zhu *et al.*^23^ prepared plate-like, hollow flower-like, and flower-cluster-like hydroxyapatite (HAP) using CaCl_2_ and (NH_4_)_2_HPO_4_ as the raw materials and SDS as an additive. The exposed negatively charged phosphate groups on HAP enhanced the adsorption capacity for cationic dye MB. Meira *et al.*^24^ prepared a magnetic magnesium–aluminum layered double hydroxide (Mag-MgAl) and modified its surface with SDS surfactant. The results showed that Mag-MgAl did not interact with MB, whereas Mag-MgAl/SDS successfully removed MB, indicating that SDS acted as a nanoreactor to attract cationic pollutants. Clearly, materials surface-modified with SDS as a modifier can effectively remove pollutants from the environment. However, the activated carbon, hydroxyapatite, and magnesium–aluminum layered double hydroxide used in the above-mentioned studies have the disadvantage of high cost, and thus not conducive to large-scale production. Therefore, considering the low cost and wide availability of fly ash as solid waste, its use as a silicon source with a silicon dioxide content of over 50% for modification can effectively improve its adsorption performance and achieve high-value applications.

Therefore, based on the high silicon content and adsorption characteristics of fly ash, two novel adsorbents, mesoporous silicon-based materials (MSM) and sodium dodecyl sulfate modified fly ash (SDS-FA), were prepared *via* an ultrasound-assisted, alkali fusion-hydrothermal method and surface modification method. Furthermore, the effects of adsorbent dosage, initial pH, contact time, and initial concentration of the solution on the adsorption of the organic pollutant MB by fly ash, MSM, and SDS-FA were investigated to select the optimal modified high silicon fly ash adsorbent. The adsorption mechanisms of MB on MSM were elucidated through adsorption isotherms, adsorption kinetics, and characterization techniques such as SEM, XRD, FTIR, and BET. By applying fly ash in the preparation of MSM, this study provides a technical reference for its comprehensive utilization.

## 2 Materials and methods

### 2.1 Materials and reagents

The fly ash used in this study was sourced from Fuxin City, Liaoning Province, China (42.01°N, 121.65°E). The fly ash was sieved to obtain particles with a size in the range of 0.25–0.38 mm. Subsequently, they were washed three times with deionized water and dried in a blast drying oven (DHG-9030, Shanghai Yiheng Scientific Instrument Co., Ltd, China) at 378.15 K for further use. The main chemical composition of the fly ash is shown in Table 1.

**Table 1:** Main compositions of Fuxin fly ash

Constituent	SiO_2_	TiO_2_	Al_2_O_3_	Fe_2_O_3_	MnO	MgO	CaO	Na_2_O	K_2_O	P_2_O_5_
Content (%)	67.10	0.12	19.74	3.35	0.34	2.87	4.00	1.08	1.30	0.10

The required reagents for the experiment are as follows: NaOH, HNO_3_, sodium dodecyl sulfate, ethanol, and methylene blue. All the above-mentioned reagents were of analytical grade and obtained from China National Pharmaceutical Group Chemical Reagent Co., Ltd (Shanghai, China). Deionized water was used throughout the experimental process.

#### 2.1.1 Simulating MB wastewater

MB powder was dissolved in deionized water to prepare an MB stock solution with a concentration of 1000 mg L^−1^ by calculating the appropriate ratio. Subsequently, the stock solution was diluted with deionized water to obtain solutions with different concentrations as needed for the experiment. The pH of the solutions was adjusted using 0.1 mol per L HNO_3_ and 0.1 mol per L NaOH.

### 2.2 Preparation of MSM and SDS-FA

#### 2.2.1 MSM

Fly ash and NaOH were uniformly mixed in a crucible in a mass ratio of 1 : 1.2. Subsequently, the mixture was subjected to high-temperature calcination at 823.15 K for 1 h in a box-type muffle furnace (KSL-1700X-A1, Hefei Kejing Co., Ltd, China) and cooled to room temperature (298.15 K). The resulting product was ground to a particle size in the range of 0.25–0.38 mm and mixed with deionized water. The obtained mixture was stirred using a magnetic stirrer (84-1, Suzhou Guohua Instrument Co., Ltd, China) at 300 rpm for 2 h. Subsequently, it was transferred to an ultrasonic cleaning machine (YM-031S, Shandong Jiajing Medical Technology Co., Ltd, China) and subjected to ultrasonic treatment in a water bath at 40 kHz and 240 W for a specific period. The mixture was allowed to age at 298.15 K for 20 h. Next, the aged white gel was transferred to a 100 mL stainless steel alloy high-pressure reactor (CY-03628, Xi'an Changyi Manufacturing Co., Ltd, China) and reacted at 373.15 K for a certain duration. Subsequently, the reaction mixture was cooled to 298.15 K and the product washed with deionized water until the pH reached 8. Finally, the product was dried in a blast drying oven (DHG-9030, Shanghai Yiheng Scientific Instrument Co., Ltd, China) at 353.15 K, followed by grinding, sieving, and storage for future use.

#### 2.2.2 SDS-FA

10 g of fly ash with a particle size of 0.25–0.38 mm was weighed in a beaker and 200 mL of deionized water added for dispersion. Then, the beaker was placed in an ultrasonic cleaning machine (YM-031S, Shandong Jiajing Medical Technology Co., Ltd, China) and heated in a water bath at 333.15 K. 3 g of SDS was added and the mixture was subjected to ultrasonic treatment at 40 kHz and 240 W for 15 min. At 298.15 K, it was transferred to a constant temperature oscillator (SHA-8, Jiangsu Guowang Instrument Co., Ltd, China) and oscillated for 8 h. After, the mixture was allowed to settle, the supernatant poured off, and the product washed with deionized water until neutral. Then, it was washed with 50% ethanol and centrifuged at 3000 rpm for 5 min using a benchtop centrifuge (TD5A-WS, Shanghai Jinghe Analytical Instrument Co., Ltd, China). Finally, the product was dried in a blast drying oven (DHG-9030, Shanghai Yiheng Scientific Instrument Co., Ltd, China) at 368.15 K, followed by grinding, sieving, and storage for future use.

### 2.3 Performance evaluation test method

To determine the application potential of the modified high silica fly ash as an adsorbent, the adsorption performances of MSM and SDS-FA were compared. Different factors were studied in the batch mode, including adsorbent dosage, initial pH, contact time, and initial concentration, to investigate their effects on the removal of MB by MSM and SDS-FA. The control group was set by adding an equivalent amount of fly ash with a particle size of 0.25–0.38 mm to the wastewater. Three parallel experiments were conducted, and the average values were taken as the final measured results. The removal efficiency (*η*, %) and adsorption capacity (*q*, mg g^−1^) were calculated using the following equations:1
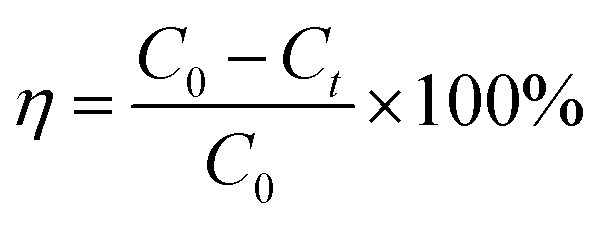
2
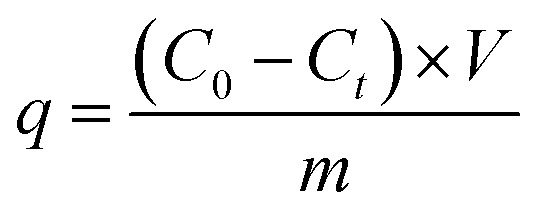
where *η* is the removal efficiency, %; *C*_0_ is the initial concentration, mg L^−1^; and *C*_*t*_ is the remaining concentration, mg L^−1^. *m* is the mass of adsorbent, g.

#### 2.3.1 Experimental procedure

At 298.15 K, under the condition of an initial pH of 9 and an initial concentration of MB of 100 mg L^−1^, different masses (1, 2, 3, 4, and 5 g L^−1^) of MSM and SDS-FA were added separately to 100 mL of MB simulated wastewater. The solution was stirred at a speed of 300 rpm for 150 min, and then samples were withdrawn and their concentrations measured to investigate the effect of the adsorbent dosage on the removal of MB by MSM and SDS-FA. At 298.15 K, under the condition of varying initial pH value (3, 4, 5, 6, 7, 8, 9, and 10) and an initial MB concentration of 100 mg L^−1^, 2 g L^−1^ of MSM and SDS-FA was added separately to 100 mL of MB simulated wastewater. The solution was stirred at a speed of 300 rpm for 150 min, and then samples were withdrawn and their concentrations measured to investigate the effect of the initial pH of the solution on the removal of MB by MSM and SDS-FA. At 298.15 K, under the condition of an initial pH of 9 and an initial concentration of MB at 100 mg L^−1^, 2 g L^−1^ of MSM and SDS-FA was added separately to 100 mL of MB simulated wastewater. The solution was stirred at a speed of 300 rpm for different contact times (5, 10, 20, 30, 40, 50, 60, 90, 120, 150, and 180 min), and then samples were withdrawn and their concentrations measured to investigate the effect of contact time on the removal of MB by MSM and SDS-FA. At 298.15 K, under the condition of an initial pH of 9 and varying initial concentrations of MB (60, 100, 140, 180, and 220 mg L^−1^), 2 g L^−1^ of MSM and SDS-FA was added separately to 100 mL of MB simulated wastewater with different MB concentrations. The solution was stirred at a speed of 300 rpm for 150 min, and then samples were withdrawn and their concentrations measured to investigate the effect of the initial concentration of MB on the removal of MB by MSM and SDS-FA.

### 2.4 Adsorption kinetics and adsorption isotherm test method

#### 2.4.1 Adsorption kinetics test for MB

At 298.15 K and 300 rpm, 2 g L^−1^ of MSM and SDS-FA was separately added to 100 mL MB simulated wastewater (pH = 9, initial concentration 100 mg L^−1^) for the adsorption experiment. After stirring for 5, 10, 20, 30, 40, 50, 60, 90, 120, 150, and 180 min, the remaining MB concentration in the solution was determined. Each group of experiments was repeated 3 times, and the mean value was taken. The Lagergren first-order adsorption kinetic equation, Lagergren second-order adsorption kinetic equation and internal diffusion model were used for the linear fitting of the fly ash and MSM and SDS-FA treatment of MB. The equations are as follows:

3 ln(*q*_e_ − *q*_*t*_) = ln *q*_e_ − *k*_1_*t*

4
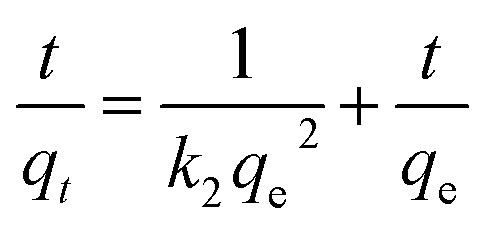

5 *q*_*t*_ = *k*_p_*t*^1/2^ + *c*

where *q*_*t*_ is the adsorption capacity at the adsorption time *t*, mg g^−1^; *q*_e_ is the equilibrium adsorption capacity, mg g^−1^; *k*_1_ is the adsorption rate constant for the pseudo-first order model, min; *k*_2_ is the adsorption rate constant for the pseudo-second order model, mg (g min)^−1^; *k*_p_ is the intraparticle diffusion rate constant, mg (g min^1/2^)^−1^; and *c* is the boundary layer related parameter, mg g^−1^.

#### 2.4.2 Adsorption isotherm test for MB

At 298.15 K and 300 rpm, 2 g L^−1^ of MSM and SDS-FA was separately added to 100 mL MB simulated wastewater (pH = 9, initial concentration 60, 100, 140, 180, and 220 mg L^−1^) for the adsorption experiments. After stirring for 150 min, the remaining MB concentration in the solution was determined. Each group of experiments was repeated 3 times and the mean value was taken. The Langmuir adsorption isotherm model and the Freundlich adsorption isotherm model were used to perform linear fitting of the adsorption of MB by fly ash, MSM, and SDS-FA employing the following equations:6
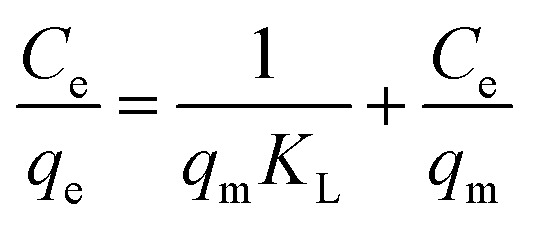
7
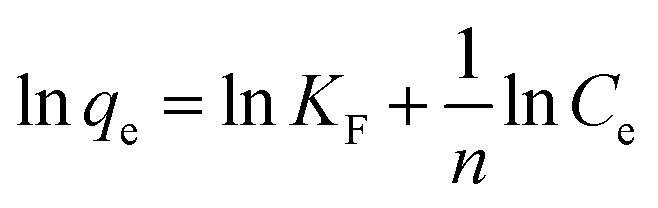
where *C*_e_ is the pollutant concentration at solution equilibrium, mg L^−1^; *q*_e_ is the adsorption capacity of the adsorbent for the pollutant at equilibrium, mg g^−1^; *q*_m_ is the saturated adsorption capacity of the adsorbent for the pollutant, mg g^−1^; *K*_L_ is the Langmuir model adsorption constant; *K*_F_ is the Freundlich model adsorption constant; and *n* is the adsorption strength correlation constant.

### 2.5 Water quality detection and material characterization method

The absorbance of MB was measured using an ultraviolet spectrophotometer (721, Shanghai Jinhe Analytical Instrument Co., Ltd, China) at a wavelength of 617 nm and the mass concentration of MB was calculated from the fitted equation of the corresponding mass concentration and absorbance. The pH was determined using the glass electrode method (China National Standard GB/T6920-86).

Scanning electron microscopy (SEM, SIGMA 500, Zeiss, Germany) was performed to analyze the changes in the micromorphology of the materials. The test conditions are as follows: current: 10 μA, voltage: 15 kV, argon gas protection, and magnification: 5000–10 000. The mineralogical composition of the material was analyzed by X-ray diffraction (XRD, D8 ADVANCE, Bruker GMBH, Germany) to determine its crystal structure. The test conditions are as follows: Cu target, voltage: 40 kV, current: 30 mA, scanning range: 2*θ* = 10–90°, step length: 0.02°, and speed: 0.5 seconds per step. Fourier transform infrared spectroscopy (VERTEX 70, Bruker GMBH, Germany) was performed to analyze the surface functional groups in the materials. The test conditions are as follows: potassium bromide (KBr) tablet method, the sample was dried and ground to a particle size of less than 0.074 mm and mixed with a KBr tablet, and scanning wavenumber range: 400–4000 cm^−1^. The Brunauer–Emmett–Teller (BET) method was used to measure the specific surface area and pore diameter of the material using a Mack ASAP 2460 (Micromeritics Instrument Ltd, USA). The test conditions are as follows: degassing time: 360 min, degassing temperature: 200 °C, test time: 360 min, and the gas was nitrogen.

### 2.6 Reusability test

At the optimal pH, contact time, and initial MB concentration, 2 g L^−1^ of MSM was added to an MB solution and stirred at 300 rpm for 150 min. Subsequently, the mixture was filtered, and the remaining MB concentration in the solution was measured. The adsorbed MSM was dried at room temperature. After drying, MSM was immersed in 50 mL of ethanol solution for a certain period to facilitate the desorption of MB. The above-mentioned steps were repeated until no MB was detected in the solution. Finally, MSM was washed with deionized water, dried, and employed in the next adsorption cycle. The reusability test was carried out for five cycles.

## 3 Results and discussion

### 3.1 Performance evaluation test

#### 3.1.1 The effect of adsorbent dosage

**Fig. 1:**
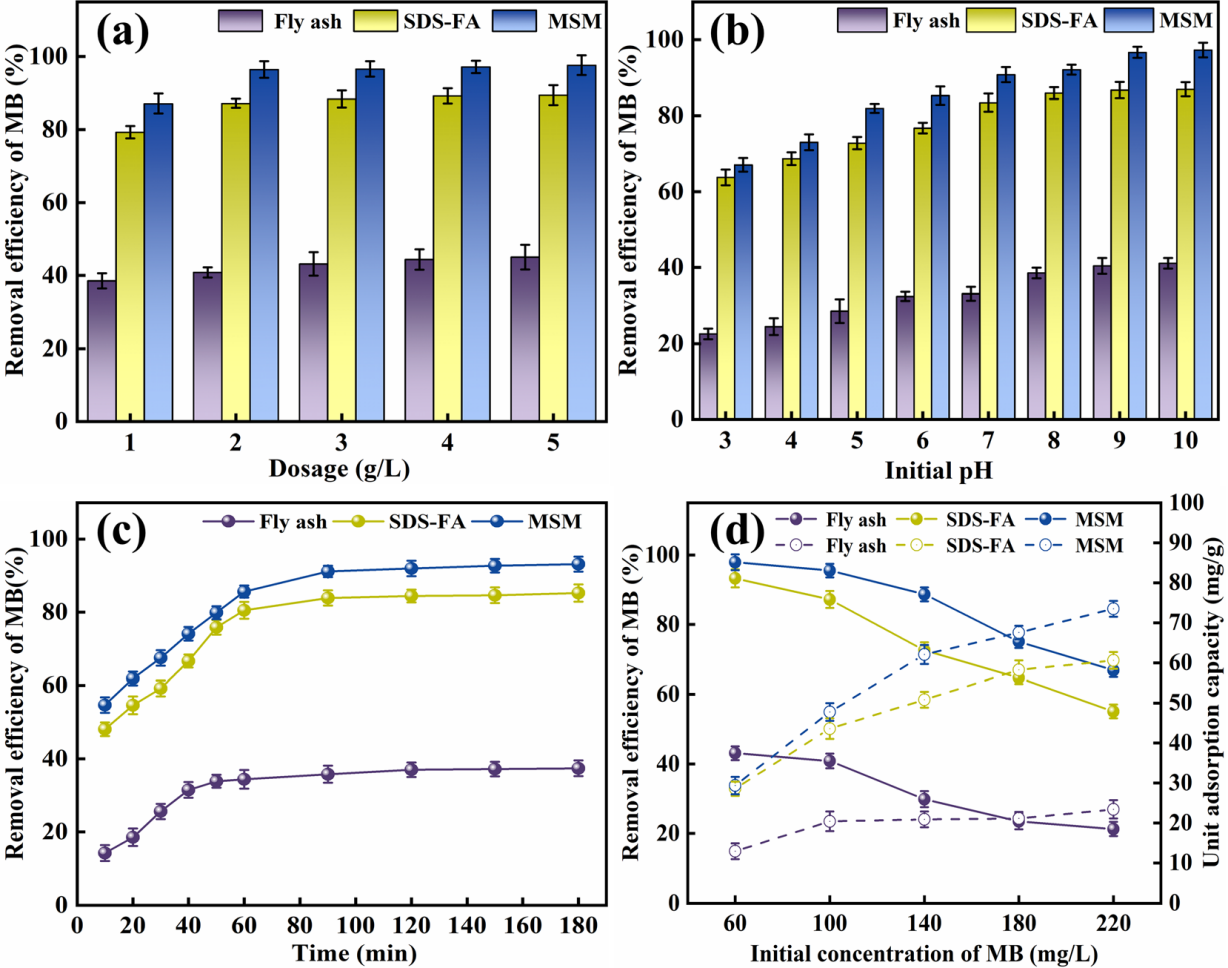
Batch experiments of the effects of different factors on the removal of MB by fly ash, MSM and SDS-FA. (a) Effect of adsorbent dosage (initial pH: 9, contact time: 150 min, initial concentration of MB: 100 mg L^−1^, temperature: 298.15 K, and stirring speed: 300 rpm). (b) Effect of initial pH (adsorbent dosage: 2 g L^−1^, contact time: 150 min, initial concentration of MB: 100 mg L^−1^, temperature: 298.15 K, and stirring speed: 300 rpm). (c) Effect of contact time (adsorbent dosage: 2 g L^−1^, initial pH: 9, initial concentration of MB: 100 mg L^−1^, temperature: 298.15 K, and stirring speed: 300 rpm). (d) Effect of initial concentration of MB (adsorbent dosage: 2 g L^−1^, contact time: 150 min, initial pH: 9, temperature: 298.15 K, and stirring speed: 300 rpm).

According to Fig. 1(a), it can be observed that MSM and SDS-FA exhibited a higher removal efficiency for MB compared to the untreated fly ash at different dosages. This indicates that the modification process effectively enhanced the adsorption performance of the materials. When the dosage of fly ash increased from 1 g L^−1^ to 5 g L^−1^, the removal efficiency of MB only slightly increased from 38.53% to 45.03%. This is because the adsorption capacity of fly ash itself is limited. SDS-FA showed a significant improvement in adsorption capacity compared to fly ash at a dosage of 1 g L^−1^, with a removal efficiency of 79.32%, which is much higher than that of fly ash at a dosage of 5 g L^−1^. This suggests that SDS modification of fly ash greatly enhanced its adsorption capacity. Subsequently, with an increase in dosage, the removal efficiency of SDS-FA gradually increased from 79.32% to 89.39%, and then stabilized. MSM exhibited a removal efficiency of 87.14% for MB at a dosage of 1 g L^−1^, which is also higher than the maximum removal efficiency of the untreated fly ash. This indicates that the mesoporous structure of the material has high adsorption potential. With an increase in dosage, the removal efficiency of MSM gradually increased from 87.14% to 97.61%, and then stabilized. According to the comparison of the adsorption performance of MSM and SDS-FA for MB, it can be concluded that SDS-FA showed a significant improvement due to the introduction of SDS molecules, which increased the hydrophobicity of fly ash and provided more electrostatic adsorption sites, thereby enhancing the adsorption of dye molecules.^25^ Alternatively, the modification effect of MSM was greater, which can be attributed to its mesoporous structure providing abundant adsorption sites and good diffusion channels for the dye molecules. Additionally, the pore size distribution and surface chemical properties of mesoporous silica-based materials also play a crucial role.^26^ Thus, considering these factors, a dosage of 2 g L^−1^ was used for subsequent experiments.

#### 3.1.2 The effect of initial pH

According to Fig. 1(b), it can be observed that under acidic conditions, MSM and SDS-FA exhibited a poor adsorption performance for MB. This is because MB is a cationic dye with a positive charge on its surface, and in acidic solutions with a high concentration of H^+^, H^+^ and MB compete for the adsorption sites. Therefore, acidic solution is not favorable for the adsorption of MB by MSM and SDS-FA. As the pH of the solution increased, the removal efficiency for MB by SDS-FA gradually increased. When the initial pH of the solution increased from 3 to 7, the removal efficiency increased from 63.77% to 83.46%. This indicates that the introduction of SDS molecules significantly enhanced the adsorption performance of the material. However, when the pH increased from 8 to 10, the removal efficiency only slightly increased from 85.94% to 86.94%, remaining relatively stable. This may be due to the increased charge density of the sulfate head of the SDS molecules at high pH values, leading to the weakening of the electrostatic attraction between SDS and MB.^27^ As the solution pH increased, the removal efficiency for MB by MSM rapidly increased. When the initial pH of the solution increased from 3 to 10, the removal efficiency increased from 67.07% to 97.26%. Overall, MSM exhibited a better removal efficiency at higher pH values. This is because the acidic Si–OH groups on the surface of the mesoporous material lose H^+^ and become negatively charged under alkaline conditions, providing adsorption sites for MB. Additionally, as the pH increases, the negative charge increases, enhancing the electrostatic interaction between MSM and the dye, thereby improving the removal efficiency. Furthermore, the alkaline environment facilitates the generation of monovalent organic cationic quaternary amine salt ionic groups, which can easily interact with the hydroxyl groups on the surface of MSM.^28^ According to the comparison of the adsorption performance of MSM and SDS-FA for MB, it can be concluded that MSM maintained a high-efficiency adsorption performance under different pH conditions, demonstrating its wide applicability. Specifically, in industrial wastewater treatment processes that require rapid treatment or are sensitive to pH changes, MSM may be a preferred choice. Thus, considering these factors, pH 9 was used for subsequent experiments.

#### 3.1.3 The effect of contact time

According to Fig. 1(c), it can be observed that the removal efficiency of fly ash for MB increased slowly with an increase in contact time, and then reached a stable state. It increased from 14.20% at 10 min to 37.16% at 150 min. This indicates that the adsorption process of fly ash is relatively slow, which is possibly due to its limited specific surface area and active sites. In comparison, SDS-FA showed a significantly higher removal efficiency than fly ash, increasing from 48.08% at 10 min to 84.64% at 150 min, demonstrating its higher adsorption efficiency. This phenomenon can be attributed to the enhanced hydrophobicity of the SDS-FA surface, which improves the affinity for hydrophobic organic dyes. The removal efficiency of MSM increased from 54.67% at 10 min to 92.69% at 150 min, indicating that its porous structure provided more adsorption sites and better pore channels for MB molecules. After 150 min of contact time, the growth rate of the removal efficiency decreased, and gradually stabilized. This is because the binding process between MB and the adsorption active sites or functional groups on the adsorbent gradually became saturated and the adsorption rate is controlled by the migration rate of the dye from the outer pores to the inner pores of the adsorbent particles.^29^ According to the comparison of the adsorption performance of MSM and SDS-FA for MB, it can be concluded that MSM exhibited a higher efficiency in the removal of MB dye and could achieve its effective removal more rapidly. This is due to its porous structure, which provides more adsorption sites and better pore channels for MB molecules. Thus, considering these factors, a contact time of 150 min was used for subsequent experiments.

#### 3.1.4 The effect of initial concentration

According to Fig. 1(d), it can be observed that when the initial concentration of MB increased from 60 mg L^−1^ to 220 mg L^−1^, the removal efficiency of MSM and SDS-FA for MB decreased. In the case of MSM, its removal efficiency decreased from 97.92% to 66.83% and its adsorption capacity increased from 29.37 mg g^−1^ to 73.52 mg g^−1^. In the case of SDS-FA, its removal efficiency decreased from 93.29% to 55.08% and its adsorption capacity increased from 28.58 mg g^−1^ to 60.59 mg g^−1^. This is because with an increase in MB concentration in the solution, the concentration difference between MB in the solution and on the surface of the adsorbent increases, resulting in a greater driving force for mass transfer, which promotes the collision between MB and the functional groups on the adsorbent surface. However, at high MB concentrations, due to the lack of effective active sites, the removal efficiency of MSM and SDS-FA for MB decreased with an increase in initial MB concentration, and the increase in unit adsorption capacity gradually slowed down. Compared to the untreated fly ash, SDS-FA modified by SDS surfactant possessed more negatively charged adsorption sites due to the sulfate functional groups on SDS-FA, which provide favorable ion exchange sites for MB adsorption, enabling SDS-FA to more effectively remove the cationic dye MB.^30^ Alternatively, MSM, with its porous structure and larger specific surface area, is more conducive to the approach of dye molecules to the adsorption sites, where intermolecular forces (van der Waals forces and hydrogen bonds) play a role.^31^ The comparative analysis of the adsorption performance of MSM and SDS-FA for MB indicates that MSM exhibited a higher efficiency and suitability for the removal of MB at different concentrations. Based on the above-mentioned experiments, it can be concluded that at an initial concentration of 100 mg L^−1^, MSM and SDS-FA can effectively utilize their pore structure and modified characteristics to achieve the efficient removal of dyes. Additionally, employing 100 mg L^−1^ as the initial concentration also helps simulate the conditions of actual industrial wastewater treatment, making the experimental results more applicable. Thus, considering all the above-mentioned factors, an initial concentration of 100 mg L^−1^ of MB was used in the subsequent experiments.

### 3.2 Adsorption kinetics and adsorption isotherm test

#### 3.2.1 Adsorption kinetics

**Fig. 2:**
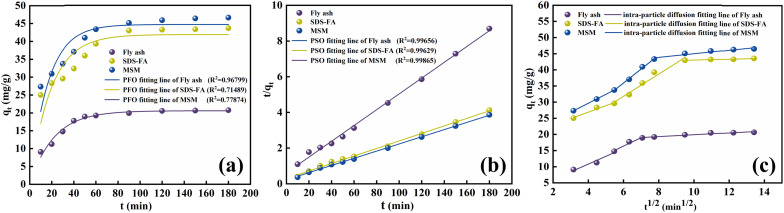
Fitting diagram of kinetic models for adsorption of MB by fly ash, MSM and SDS-FA. (a) PFO kinetic model for the adsorption of MB. (b) PSO kinetic model for the adsorption of MB. (c) Intra-particle diffusion model for the adsorption of MB (adsorbent dosage: 2 g L^−1^, initial pH: 9, initial concentration of MB: 100 mg L^−1^, temperature: 298.15 K, and stirring speed: 300 rpm).

By fitting the adsorption kinetic models, the influence of contact time on the removal of MB can be explored to understand the potential controlling steps and adsorption mechanisms during the adsorption process. Accordingly, the pseudo-first-order kinetic model (PFO), pseudo-second-order kinetic model (PSO), and intra-particle diffusion model^32,33^ were employed to analyze the adsorption kinetics of MSM and SDS-FA for MB. The fitting results are shown in Fig. 2 and Table 2.

**Table 2:** Kinetic parameters of MB adsorption by fly ash, MSM and SDS-FA

Kinetic model	Parameters	Fly ash	MSM	SDS-FA
PFO model	*k*_1_	0.04618	0.06099	0.05252
	*q*_e_	20.69792	46.57632	43.63057
	*R* ^2^	0.96799	0.77874	0.71489
PSO model	*k*_2_	3.2124 × 10^−2^	1.8076 × 10^−2^	1.5196 × 10^−2^
	*q*_e_	22.59380	49.77601	47.43833
	*R* ^2^	0.99656	0.99865	0.99629
Intra-particle diffusion model	*k*_p1_	2.41511	2.76896	1.99315
	*R* ^2^	0.91017	0.99999	0.94106
	C_1_	1.16115	18.57657	18.93932
	*k*_p2_	2.61659	4.31921	3.44228
	*R* ^2^	0.91639	0.99286	0.95863
	C_2_	0.68922	10.01584	11.15100
	*k*_p3_	0.29371	0.56496	0.16368
	*R* ^2^	0.92586	0.90106	0.94723
	C_3_	16.98034	39.34482	41.39157

According to the comparison of the *R*^2^ values of the kinetic models, it can be observed that the adsorption of MB by MSM and SDS-FA is best described by the PSO kinetic model. This indicates that the adsorption process is mainly governed by chemical adsorption. The relatively low value of *k*_1_ for fly ash is attributed to its limited specific surface area and number of active sites. Alternatively, the higher values of *k*_1_ for MSM and SDS-FA indicate their ability to adsorb dye molecules more rapidly. The modification of SDS-FA increased the number of surface active sites, thereby enhancing the adsorption rate of dye molecules. Meanwhile, the mesoporous structure of MSM provided more channels for the rapid transport and adsorption of dye molecules. The higher values of *k*_2_ for MSM and SDS-FA indicate that more chemical reaction steps are involved during the adsorption process. This further confirms that the interaction between the dye molecules and the adsorbent surface not only involves physical adsorption but also chemical adsorption. The fitting curves of the intra-particle diffusion model do not pass through the origin, and the higher values of *k*_p_ for MSM and SDS-FA indicate the significant contribution of internal diffusion to the adsorption rate. Thus, the adsorption of MB by MSM and SDS-FA is controlled by both surface diffusion and pore diffusion mechanisms. MSM and SDS-FA act as porous media, and the adsorption of MB on MSM and SDS-FA from the liquid phase involves three stages, indicating a continuous segmented process of adsorption. The first stage is mainly surface adsorption, where MB diffuses from the solution to the outer surface of MSM and SDS-FA, primarily occurring through micropore diffusion. The second stage is intra-particle diffusion, where MB adsorbs on the surface of MSM and SDS-FA and enters their internal pores. The third stage is the dynamic equilibrium stage of adsorption and desorption, where the diffusion rate decreases, gradually reaching an equilibrium state.

#### 3.2.2 Adsorption isotherm

**Fig. 3:**
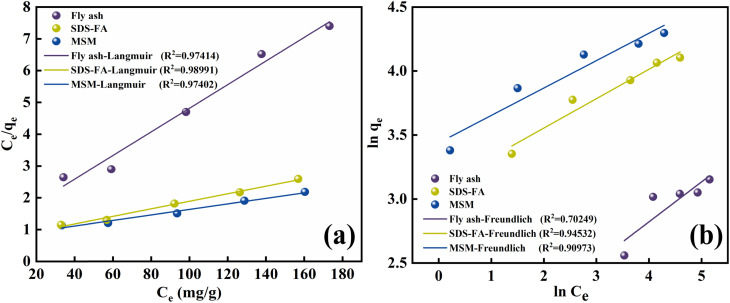
Isotherm model fitting diagram of adsorption of MB by fly ash, MSM and SDS-FA. (a) Langmuir isotherm model. (b) Freundlich isotherm model (adsorbent dosage: 2 g L^−1^, initial pH: 9, contact time: 150 min, initial concentration of MB: 100 mg L^−1^, temperature: 298.15 K, and stirring speed: 300 rpm).

Adsorption isotherms primarily describe the adsorption equilibrium relationship between an adsorbent and adsorbate at the solid–liquid interface. The Langmuir and Freundlich adsorption isotherm models^34,35^ were used to analyze the adsorption isotherms of MSM and SDS-FA for MB, and the fitting results are shown in Fig. 3 and Table 3.

**Table 3:** Isotherm parameters of MB adsorption by fly ash, MSM and SDS-FA

Isotherm model	Parameters	Fly ash	MSM	SDS-FA
Langmuir model	*q*_m_	26.91790	115.34025	84.45945
	*K*_L_	0.04080	0.00662	0.00833
	*R* ^2^	0.97414	0.97402	0.98991
Freundlich model	*n*	3.20379	4.67093	4.35369
	*K*_F_	4.84800	31.68000	22.04000
	*R* ^2^	0.70249	0.90973	0.94532

According to the fitting curves and experimental results, it can be observed that with an increase in the initial dye concentration, the equilibrium adsorption capacity, *q*_e_, of the modified fly ash-based materials also increased. This indicates that at high concentrations, there is a more frequent interaction between the dye molecules and the adsorbent, thereby promoting the adsorption process. According to the comparison of the fitting performance of the two models, the Langmuir model is better suited to describe the adsorption behavior of MSM and SDS-FA for MB, indicating that their adsorption characteristics tend towards monolayer adsorption. The values of the adsorption intensity-related coefficient, *n*, are all greater than 1, indicating that the adsorption process is spontaneous. As the adsorption sites are gradually occupied, the adsorption rate decreases until equilibrium is reached. The magnitude of the Freundlich constant, *K*_F_, is directly proportional to the adsorption capacity and adsorption intensity. The *K*_F_ value for the unmodified fly ash is 4.85, while that for SDS-FA and MSM is 22.04 and 31.68, respectively. This indicates that the modification effectively enhanced the capability of fly ash to treat dye wastewater. Additionally, the maximum adsorption capacity of MSM for MB was 115.34 mg g^−1^, while that for SDS-FA was 84.46 mg g^−1^. Compared to SDS-FA, MSM exhibited a higher efficiency for the adsorption of MB. Furthermore, the comparison with previously reported modified adsorbents in the literature demonstrates that MSM has a higher adsorption capacity and shorter adsorption equilibrium time, as shown in Table 4.

**Table 4:** Adsorption properties of different modified adsorbents for MB

Adsorbents	pH	Dosage (g L^−1^)	Equilibrium time (h)	*q*_m_ (mg g^−1^)	References
ZSM-5 zeolite	11	1.6	2	5.422	36
Rice straw with alkali-fused fly ash	7	1	15	131.58	37
Denatured fly ash	7	4	2	28.65	38
MS-CFA	9	0.5	0.5	10.86	39
Magnetic zeolites	7	6.25	0.5	27.05	40
Olive stones	9	2.8	1.5	44.5	41
Lemongrass leaves biowaste	9	10	0.75	43.16	42
SDS-FA	9	2	2.5	84.46	This study
MSM	9	2	2.5	115.34	This study

### 3.3 Characterization results

#### 3.3.1 SEM

**Fig. 4:**
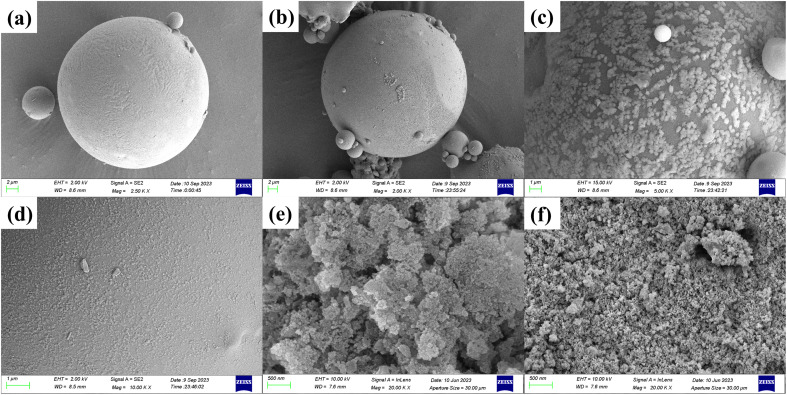
SEM images of fly ash, SDS-FA and MSM before and after adsorption of MB. Fly ash (a) before and (b) after adsorption. SDS-FA (c) before and (d) after adsorption. MSM (e) before and (f) after adsorption.

To examine the surface microstructure of fly ash, SDS-FA, and MSM, SEM was used to observe the sample surfaces, and the results are shown in Fig. 4. According to Fig. 4(a) and (b), it can be observed that the surface structure of fly ash before adsorption is relatively uniform and it is a smooth sphere. After adsorption, its surface roughness significantly increases, and a large amount of particle-like deposits appear. This is due to the interaction between the MB dye molecules and certain active sites on the surface of fly ash, leading to the deposition of the dye molecules on the fly ash surface. Fig. 4(c) and (d) show that numerous SDS flake crystal fragments are uniformly attached to the SDS-FA surface before adsorption, indicating that SDS was successfully coated on the fly ash particles. After adsorption, uniform particle-like precipitates appeared on the surface of SDS-FA, and its structure becomes denser. This is because of the electrostatic interaction between the SDS molecules on the surface of SDS-FA and the dye molecules, resulting in the redistribution of the SDS molecules on the fly ash surface and the formation of a more uniform layered structure, making the originally rough surface smoother.^43^

Fig. 4(e) and (f) demonstrate that compared to fly ash, the surface of MSM became rougher and exhibited a loose honeycomb-like structure with distinct crystallinity. The presence of pore structures between the grains is evident, and the pores appear well-developed. This is attributed to the corrosion effect of the alkali solution, which led to the formation of cavities and voids on the surface. The alkali melt residue after ultrasonic treatment further enriched the products. In the hydrothermal reaction kettle, the glass phase in fly ash was destroyed, resulting in a rougher surface and a blocky porous structure. The honeycomb-like structure of MSM collapsed to different degrees after the reaction and the number of pore structures decreased. This is due to the occupation of some of the pores on the MSM surface by dye molecules or complexes formed during the adsorption process. Additionally, the adsorption of dye molecules may cause changes in the silicate structure on the surface or inside MSM, thereby affecting the stability of its pore structure.^44^

#### 3.3.2 XRD

**Fig. 5:**
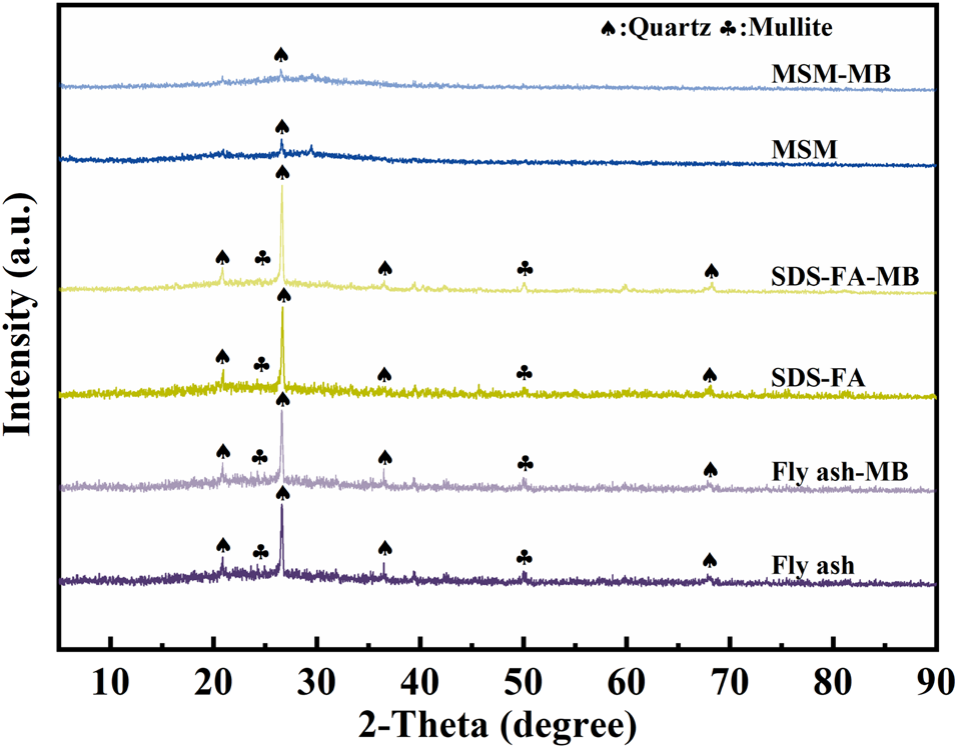
X-ray diffraction patterns of fly ash, SDS-FA and MSM before and after the adsorption of MB.

The XRD results for fly ash, SDS-FA and MSM are shown in Fig. 5. Fly ash contains crystalline and amorphous glassy substances, and quartz (PDF: #83-2465) diffraction peaks appeared at 2*θ* values of 20.76°, 26.56°, and 40.62°, and mullite (PDF: #84-1205) diffraction peaks appeared at 25.67° and 50.32°.^45^ The wide bulging peaks in the figure indicate the presence of an amorphous glass phase in the fly ash, mainly amorphous SiO_2_, indicating that it can provide a good silicon source for the synthesis of mesoporous silicon-based materials. After adsorption, the relative intensity of the diffraction peaks of mullite and quartz of fly ash was weakened, which is due to the strong acid and alkaline resistance of the crystal phases such as mullite and quartz, which are not easily dissolved.^46^ After surface modification with SDS, the characteristic peaks of mullite and quartz in the fly ash did not weaken, and the positions of the diffraction peaks remained almost unchanged. This indicates that the modification did not disrupt the crystal structure of the fly ash and the main component of the modified material was still fly ash. This is because SDS molecules do not possess a crystalline structure, and the modification process does not generate new crystal phases. The relative intensities of the quartz and mullite diffraction peaks in MSM decreased, indicating that the Si–O tetrahedra of quartz and mullite were broken down into silicon oxide molecules during the preparation of MSM. Simultaneously, the silicon oxide molecules aggregated into larger particles and stacked, resulting in an increase in pore size and the formation of a porous structure. This also indicates the successful preparation of the mesoporous silicon-based materials. The wide bulging peaks in the diffraction pattern in the range of 2*θ* = 21.27–32.91° shows a decrease in the diffraction peak, indicating the destruction of the amorphous SiO_2_ glass phase and a rougher surface structure, which is consistent with the SEM characterization results. This also suggests that the mesoporous silicon-based material is in an amorphous state. According to the comparison of the XRD diffraction peaks before and after adsorption, it can be concluded that the influence of the adsorption process on the crystal structure of fly ash, SDS-FA, and MSM was negligible.

#### 3.3.3 FTIR

**Fig. 6:**
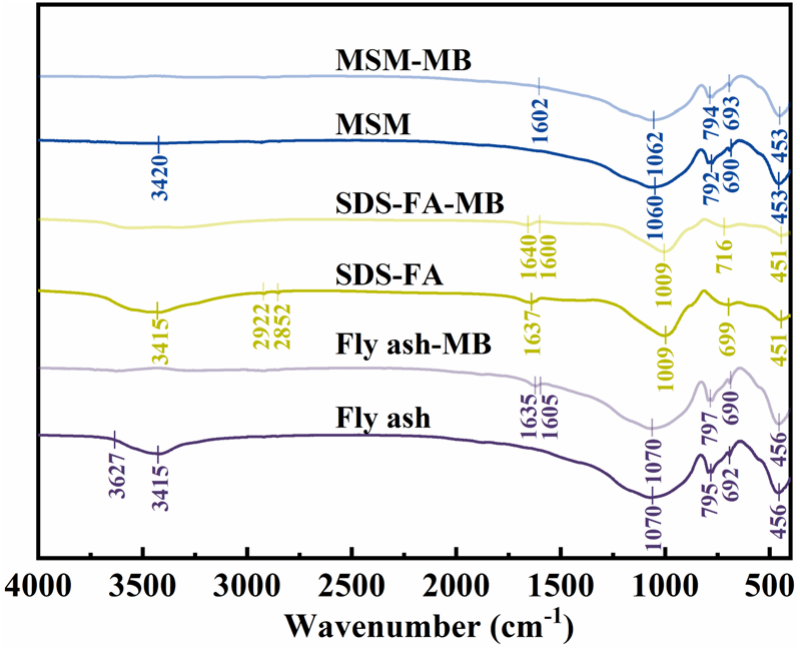
FT-IR spectra of fly ash, SDS-FA and MSM before and after the adsorption of MB.

The FTIR results for fly ash, SDS-FA and MSM are shown in Fig. 6. The absorption peak in the spectrum of fly ash at 456 cm^−1^ is attributed to the bending vibration of the skeleton Si–O bond, while the absorption peak at around 795 cm^−1^ corresponds to the Si–O–Si symmetric stretching vibration peak of the SiO_4_ tetrahedron. The absorption peak at 962 cm^−1^ is attributed to the bending vibration of Si–OH. Also, the absorption peak at 1070 cm^−1^ is Si–O–Si attributed to the antisymmetric stretching characteristic peak. The wider absorption peak at around 3415 cm^−1^ corresponds to the characteristic peak of the O–H stretching vibration in Si–OH and interlayer water. The S–O tensile vibration at 900–1000 cm^−1^ and the C–H asymmetric and symmetrical stretching vibration peaks at 2922 cm^−1^ and 2852 cm^−1^, respectively, correspond to the alkyl characteristic peaks of SDS. Thus, the results show that the SDS molecules entered the fly ash or covered its surface, and thus the sodium dodecyl sulfate-modified fly ash material was successfully prepared. The hydroxyl stretching vibration peak of water molecules at around 3415 cm^−1^ was weakened, which is because the water molecules between the fly ash layers were extruded after SDS entered the interlayer, and the interlayer water content decreased obviously. Simultaneously, the hydrophobicity of SDS-FA increased. The absorption peak of Si–O–Mg at 676 cm^−1^ disappeared for MSM, indicating that MSM destroyed the surface crystals through chemical bonding in Si–O–Mg and a pore structure was formed between the grains, which is consistent with the XRD results. After the adsorption of MB by fly ash, SDS-FA and MSM, the characteristic absorption peaks of the aromatic rings belonging to the MB dye appeared at the wavenumber of around 1600 cm^−1^, indicating that MB was adsorbed on the surface or in the voids, and the adsorption process occurred. The intensity of the absorption peak at 1062 cm^−1^ for MSM was weakened, indicating that some silicates in MSM were dissolved and the Si–O bonds were broken, proving that Si–O–Si played a role in the adsorption reaction. In addition, the stretching vibration and bending vibration peaks corresponding to Si–OH and interlayer water–OH at the wavenumber of 3415 cm^−1^ were weakened or disappeared after the adsorption of MB dye, indicating that hydrogen bonds were involved in the adsorption of MB dye on the surfaces of fly ash, SDS-FA and MSM in addition to electrostatic attraction during adsorption.

#### 3.3.4 BET

**Fig. 7:**
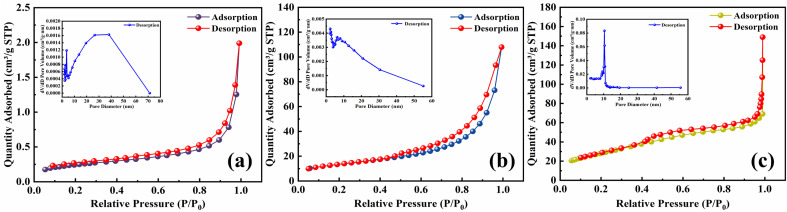
N_2_ adsorption–desorption isotherms and pore-aperture differential distribution curves of fly ash, SDS-FA and MSM. (a) Fly ash, (b) MSM and (c) SDS-FA.

The results calculated based on the BET adsorption isotherm equation and BJH method are shown in Table 5. Fig. 7 displays the N_2_ adsorption–desorption isotherms of fly ash, SDS-FA and MSM, and the inset shows the BJH pore size distribution curve. The specific surface area of SDS-FA (93.59 m^2^ g^−1^) is significantly higher than that of fly ash (0.86 m^2^ g^−1^), with an increase by 108.83 times. This is because the addition of SDS changed the surface chemical properties of fly ash, effectively improving the dispersion of the fly ash particles and reducing their aggregation, resulting in a significant increase in the specific surface area of fly ash. Additionally, the sulfate head of SDS may exhibit strong interactions with nitrogen molecules, thereby enhancing the adsorption capacity. Combined with the SEM analysis, the hydrophobic long chains of SDS likely formed a coating on the surface of fly ash, providing more adsorption sites. The specific surface area of MSM is 48.92 m^2^ g^−1^, which is 58.88 times higher than that of fly ash. According to Fig. 7, it can be observed that MSM did not show a significant difference at *P*/*P*_0_ values below 0.45, but noticeable adsorption occurred in the range of 0.45–0.9. According to the classification by Brunauer *et al.*,^47^ the isotherm type belongs to type IV, which is the typical characteristic of mesoporous materials. The adsorption observed in the range of 0.9–1.0 is attributed to the formation of voids between the MSM grains, which is consistent with the SEM analysis results. The hysteresis loop of type H1 in the pressure range of 0.65–1.0 indicates a uniform pore size and relatively narrow pore size distribution. The pore size distribution curve shows that the MSM pores are mainly concentrated in the range of 8–15 nm. The formation of pore structures in mesoporous materials is mainly due to the gradual connection of silicon–oxygen and aluminum–oxygen tetrahedra to form a framework during their preparation. Simultaneously, water molecules and other solutes enter the crystal structure, forming channels within the crystal. Clearly, the higher specific surface area of MSM is crucial for the removal of MB pollutant from water.

**Table 5:** Specific surface area and pore size of fly ash, SDS-FA and MSM

Type	Specific surface area (m^2^ g^−1^)	Pore volume (cm^3^ g^−1^)	Pore diameter (nm)
Fly ash	0.86	0.0029	13.53
SDS-FA	93.59	0.0741	10.12
MSM	48.92	0.1633	8.78

### 3.4 Reusability

**Fig. 8:**
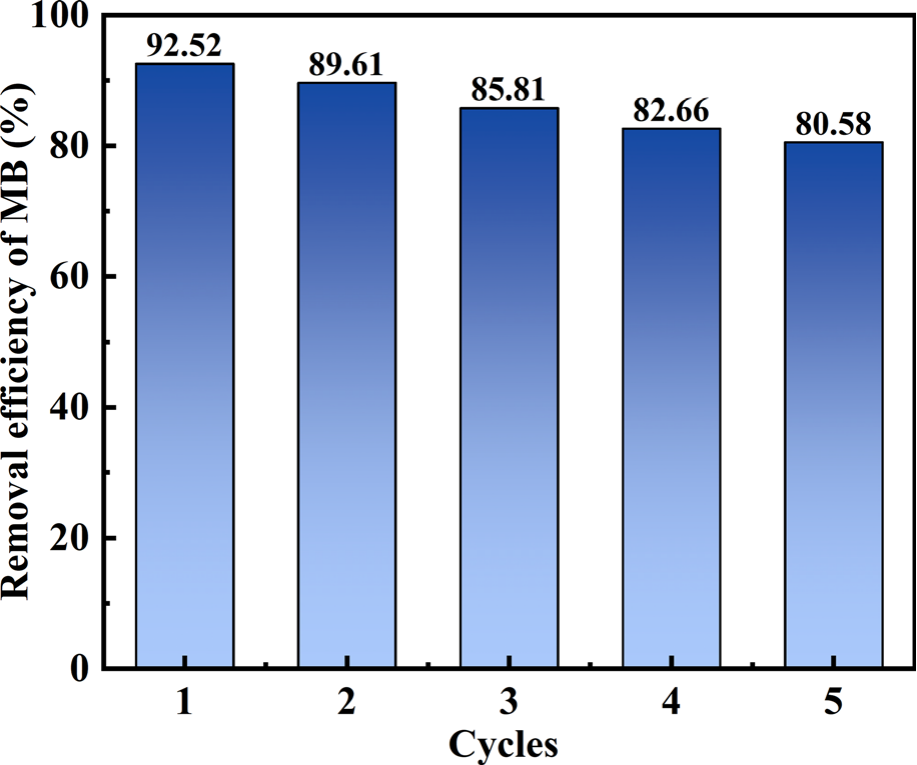
Removal efficiency during 5 cycles.

Reusability describes how an adsorbent used in the adsorption process can be regenerated using a desorption agent. Thus, the reusability of the novel adsorbent MSM was investigated, which verified its stability and regeneration ability, thereby achieving a cost-effective adsorption process and effective utilization of the adsorbent. Ethanol is an effective desorbent.^48^ The hydroxyl groups in ethanol can weaken the interaction between the adsorbent and the dye, promoting the desorption of MB. Therefore, ethanol was chosen for the cyclic adsorption experiments, and the results are shown in Fig. 8. It can be observed that after five cycles of adsorption–desorption, the removal efficiency for MB by MSM gradually decreased from the first cycle (92.52%) to the fifth cycle (80.58%). After five cycles, the removal efficiency remained above 80%, emphasizing the excellent stability and reusability of MSM and highlighting its potential application for the efficient treatment of dye-containing wastewater.

### 3.5 Adsorption mechanism

**Fig. 9:**
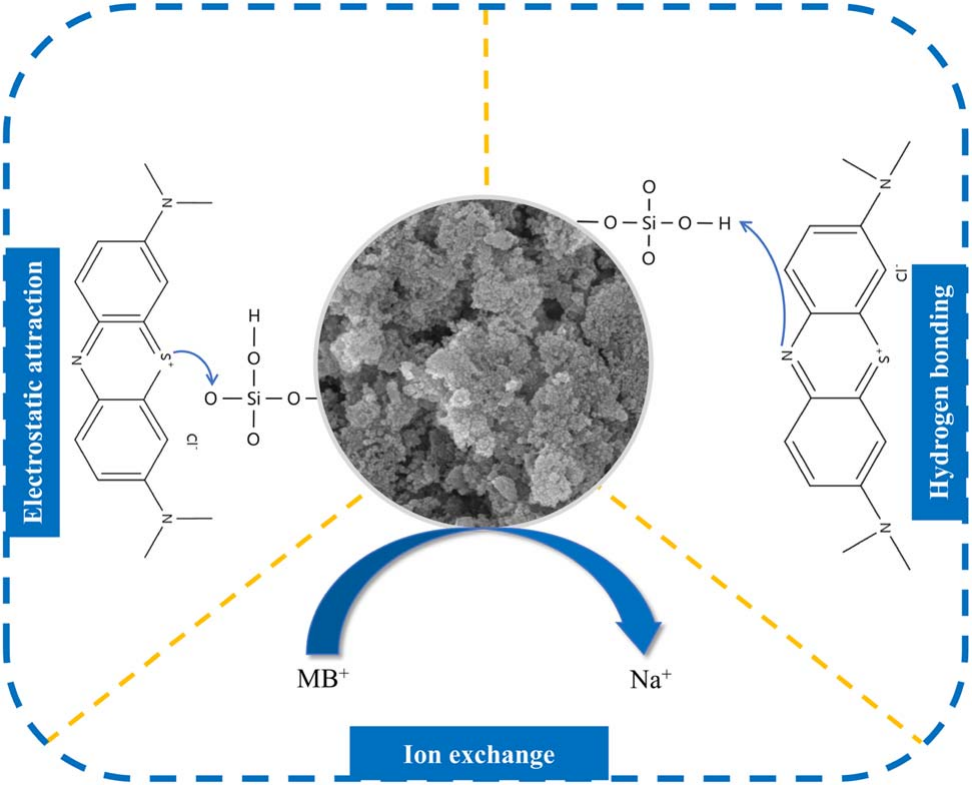
Adsorption mechanism of MSM on MB.

The adsorption mechanism of MSM on MB was predicted through performance evaluation tests, investigation of adsorption kinetics and isotherms, and characterization of MSM. MSM exhibited an excellent adsorption performance for MB, which can be explained from the perspectives of its structural characteristics and surface activity. After ultrasonication and alkali fusion activation, the surface of fly ash became rough, its structure became loose, exhibiting a honeycomb-like morphology, and its crystal structure became apparent. Pore structures were formed between the grains and a pore structure was well-developed. Compared to fly ash, MSM possessed a significantly increased specific surface area and pore volume, providing more adsorption sites. Thus, the favorable pore structure of MSM facilitated the diffusion of MB into its pores, consistent with the BET analysis results. Additionally, the interaction forces between MSM and MB were crucial for its high adsorption capacity. According to the adsorption kinetics and isotherm studies, the adsorption of MB by MSM followed the Langmuir model (*R*^2^ = 0.97402, 0.98991) and pseudo-second-order kinetic model (*R*^2^ = 0.99865, 0.99629), indicating a monolayer adsorption process dominated by chemical adsorption. This is because the hydroxyl groups on the surface of MSM can form hydrogen bonds with the nitrogen atoms on the MB molecules. The FT-IR analysis before and after adsorption supported the presence of hydrogen bonding interactions. The interconnected cavities formed by the SiO_4_ framework in MSM often contained Na^+^ and water molecules, and Na^+^ could exchange with MB^+^ in the liquid phase. MSM still exhibited certain adsorption capacity under acidic conditions, which was related to ion exchange.^49,50^ The Si–OH in MSM also played a crucial role in the adsorption of MB. This is because under neutral or alkaline conditions, these silanol groups lost a proton (H^+^) to the surrounding water (*i.e.*, deprotonation). This deprotonation led to the formation of negatively charged silicates [Si–O]^−^ on the surface of MSM, resulting in a net negative charge on its surface. Consequently, the positively charged MB^+^ was attracted to the surface of MSM through electrostatic interactions. However, under acidic conditions (low pH), the silanol groups on the surface of silica could accept protons (H^+^) from the surrounding water. This process neutralized the negative charge on the silicate groups (*i.e.*, protonation). As the pH decreased further, the negative charge on the surface of MSM gradually decreased and could even reach a neutral state, which hindered the adsorption of MB^+^ on MSM. This is consistent with the lower adsorption capacity and FT-IR characterization in acidic environments. A schematic diagram of the adsorption mechanism is shown in Fig. 9.

## 4 Conclusions

This study was based on the high silicon content and adsorption characteristics of fly ash. Two novel adsorbents, MSM and SDS-FA, were prepared using an ultrasound-assisted, alkali fusion-hydrothermal method and surface modification method. Performance evaluation tests were conducted to investigate the effects of various factors on the removal of MB by MSM and SDS-FA, aiming to optimize the modified high-silicon fly ash. The results showed that under the conditions of an adsorbent dosage of 2 g L^−1^, initial pH of 9, contact time of 150 min, and initial MB concentration of 100 mg L^−1^, the removal efficiency of MB by MSM and SDS-FA was 92.69% and 84.64%, respectively, which was significantly higher than that of fly ash. The adsorption of MB by MSM and SDS-FA followed the Langmuir model (*R*^2^ = 0.97402 and 0.98991) and pseudo-second-order kinetic model (*R*^2^ = 0.99865 and 0.99629), indicating a monolayer adsorption process dominated by chemical adsorption, respectively. The adsorption mechanism of MB by MSM was attributed to its increased specific surface area, hydrogen bonding, ion exchange, and electrostatic interactions. After five cycles of adsorption–desorption process, the removal efficiency of MB dye by MSM consistently remained above 80%. A comparison of the adsorption performance of MSM and SDS-FA revealed that MSM maintained a high adsorption performance under different pH conditions and with different concentrations of MB dye, demonstrating its wide application potential. In conclusion, MSM prepared using high-silicon fly ash as a silicon source can be utilized as a simple and effective adsorbent for the environmentally friendly removal of MB dye. Furthermore, MSM shows promising prospects for large-scale industrial applications and provides new ideas and technical support for the value-added utilization of fly ash.

## Data availability

Data will be made available on request.

## Author contributions

Xuying Guo: funding acquisition, conceptualization, methodology, validation, formal analysis, writing—original draft. Zilong Zhao: software, resources, data curation, visualization, writing—original draft preparation, writing—review and editing. Xinle Gao: resources, project administration, supervision. Yanrong Dong: funding acquisition, conceptualization, methodology, resources, supervision. Honglei Fu: project administration, supervision, validation. Xiaoyue Zhang: investigation, project administration, supervision.

## Conflicts of interest

The authors declare that they have no known competing financial interests or personal relationships that could have appeared to influence the work reported in this paper.
